# Characterization and engineering of *Streptomyces griseofuscus* DSM 40191 as a potential host for heterologous expression of biosynthetic gene clusters

**DOI:** 10.1038/s41598-021-97571-2

**Published:** 2021-09-15

**Authors:** Tetiana Gren, Christopher M. Whitford, Omkar S. Mohite, Tue S. Jørgensen, Eftychia E. Kontou, Julie B. Nielsen, Sang Yup Lee, Tilmann Weber

**Affiliations:** 1 The Novo Nordisk Foundation Center for Biosustainability, Technical University of Denmark, Kemitorvet, bygning 220, 2800 Kgs. Lyngby, Denmark; 2 Metabolic and Biomolecular Engineering National Research Laboratory, Department of Chemical and Biomolecular Engineering, Center for Systems and Synthetic Biotechnology, Institute for the BioCentury, Korea Advanced Institute of Science and Technology, Daejeon, Republic of Korea

**Keywords:** Antimicrobials, Biotechnology, Applied microbiology, Antimicrobials, Industrial microbiology, Antimicrobials, Metabolic engineering

## Abstract

*Streptomyces griseofuscus* DSM 40191 is a fast growing *Streptomyces* strain that remains largely underexplored as a heterologous host. Here, we report the genome mining of *S. griseofuscus*, followed by the detailed exploration of its phenotype, including the production of native secondary metabolites and ability to utilise carbon, nitrogen, sulphur and phosphorus sources. Furthermore, several routes for genetic engineering of *S. griseofuscus* were explored, including use of GusA-based vectors, CRISPR-Cas9 and CRISPR-cBEST-mediated knockouts. Two out of the three native plasmids were cured using CRISPR-Cas9 technology, leading to the generation of strain *S. griseofuscus* DEL1. DEL1 was further modified by the full deletion of a pentamycin BGC and an unknown NRPS BGC, leading to the generation of strain DEL2, lacking approx. 500 kbp of the genome, which corresponds to a 5.19% genome reduction. DEL2 can be characterized by faster growth and inability to produce three main native metabolites: lankacidin, lankamycin, pentamycin and their derivatives. To test the ability of DEL2 to heterologously produce secondary metabolites, the actinorhodin BGC was used. We were able to observe a formation of a blue halo, indicating a potential production of actinorhodin by both DEL2 and a wild type.

## Introduction

The vast majority of clinically used antibiotics, antifungals, anticancer and immunosuppressive drugs are derived from natural products^1^ originating from soil-inhabiting Actinobacteria^2^. Genes, necessary for the production of a particular compound, are typically arranged in biosynthetic gene clusters (BGCs) that can be detected using genome mining tools like antiSMASH^3^. With the revolution in whole genome sequencing it became clear that the majority of actinobacterial strains possess genomes of large sizes, with 20–40 BGCs on average^4^. However, typically these bacteria only produce a few compounds under laboratory conditions^5^. Other antibiotics are not produced or are only produced in small amounts and require induction by various, frequently unknown, environmental cues^5,6^. In order to unravel the biosynthetic richness of Actinobacteria, multiple genetic engineering methods, e.g. induction of BGCs by co-expression of regulatory genes^7,8^, use of transcription factor decoys^9^, refactoring of the BGCs^10,11^ or knockouts of the core genes of “constitutively” produced compounds^12^ have been explored. Recently, broader attention has been brought to the development of heterologous expression hosts for the discovery and characterization of novel metabolites. Heterologous hosts are typically well-characterized strains, that possess a plurality of needed characteristics, e.g. fast and disperse growth, amenability to genetic engineering, high yields of produced secondary metabolites^11,13,14^. The use of such chassis strains is highly advantageous, when the native producer strain can not be genetically engineered or is slow growing^13^. At the moment, several of such *Streptomyces* hosts are available, such as derivatives of *S. coelicolor*, *S. lividans*, *S. avermitilis, S. chattanoogensis, S. albus* and *S. venezuelae*^15–20^. Recently, several groups have reported the generation of *S. albus* J1074 derivatives that harbor knockouts of multiple native clusters^21,22^ and were successfully used for the expression of BGCs^23,24^. The reported success rate of BGC expression, however, remains low, reaching 30%^14^. We believe that further extending the panel of heterologous hosts will be highly beneficial to solve this challenge.

One of the promising potential heterologous hosts, *S. griseofuscus,* was first isolated in Japan and used as a host for the isolation and propagation of bacteriophages and plasmids^25–27^. Some of the strains were used for the industrial production of ε-poly-l-lysine^28–30^ and puromycin^31^, and reported as a source of novel compounds^9,32^. Even though several publications have stipulated multiple positive characteristics of* S. griseofuscus*, e.g. fast growth, ease of transformation and genetic manipulation, it was never methodically studied regarding its qualities as a potential platform for the expression of BGCs.

In this paper, we describe a comprehensive genotypic and phenotypic characterization of *S. griseofuscus* DSM40191 as a potential heterologous production host. The de novo sequenced genome of *S. griseofuscus* was used for mining of natively encoded BGCs. For the first time, we have explored the use of various genetic tools for engineering *S. griseofuscus*, ranging from the use of integrative and replicative vectors, CRISPR-Cas9 and CRISPR-BEST based knockouts, to the multiplexed-targeting of several BGCs using only one CRISPR-BEST construct. All of the generated knockouts were tested regarding their growth and production of metabolites.

## Results

### Genome mining and comparative analysis

**Figure 1:**
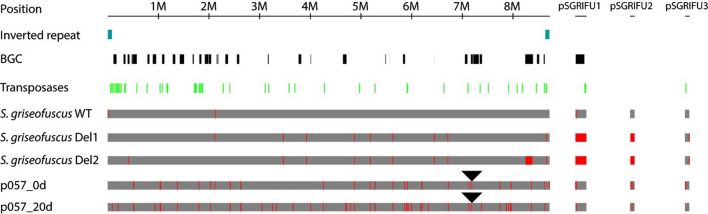
Overview of *S. griseofuscus* strains and mutations. The positions of genomic inverted repeats are highlighted in blue, BGCs in black and transposases in green. The positions of the identified mutations are highlighted in red. The mutations detected in the wild type Illumina dataset can be considered as a technical noise. The position of the CRISPR-cBEST introduced STOP-codon is indicated with a black triangle. The strains p057_0D and p057_20D relate to the long term cultivation experiment, in which CRISPR-cBEST generated strain *S. griseofuscus* IHEP81_06602 (p057_0D), that contains an introduced STOP-codon in BGC 30, was transferred 20 consecutive times in liquid ISP2 media without selective pressure, thus generating strain p057_20D. The alignment was created with CLC Genomics Workbench 12.0.3 https://digitalinsights.qiagen.com/ and visualised with Adobe Illustrator 23.0.6 https://www.adobe.com/products/illustrator.html.

*Streptomyces griseofuscus* DSM 40191 (= NRRL B-5429) was received from the German Collection of Microorganisms and Cell Cultures. We re-sequenced and assembled the genome of *S. griseofuscus *de novo^33^. It consists of a linear 8,721,740 bp chromosome and three plasmids: pSGRIFU1 (220 kb), pSGRIFU2 (88 kb), and pSGRIFU3 (86 kb) (Fig. 1).

#### Whole genome comparison between *S. griseofuscus* DSM40191, *S. coelicolor* A3(2), *S. venezuelae* ATCC 10712

**Figure 2:**
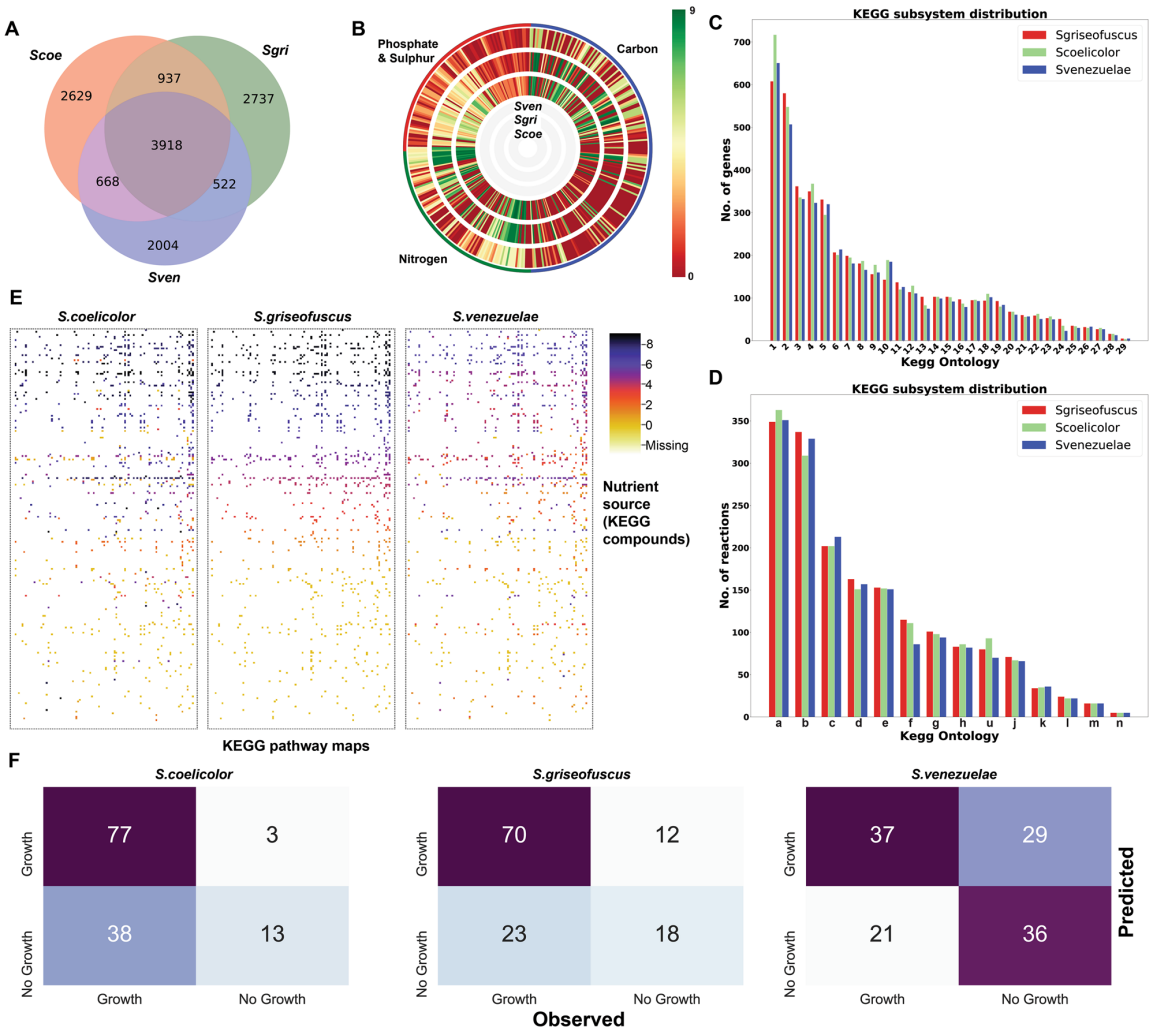
Genome and phenotype microarray comparison of three strains. (**A**) Number of genes shared among the three organisms—*S. griseofuscus* (sgri), *S. coelicolor* (scoe), *S. venezuelae* (sven). (**B**) Phenotype microarray data represented by activity index rings generated using DuctApe across 4 biolog plates consisting of 379 carbon, nitrogen, phosphate and sulphate nutrient sources across the three organisms. (**C**) Distribution of the number of genes per KEGG subsystem across the three organisms calculated using KEGG Automatic Annotation Server^35^. 1—Protein families: signalling and cellular processes; 2—Protein families: genetic information processing; 3—Protein families: metabolism; 4—Carbohydrate metabolism; 5—Amino acid metabolism; 6—Metabolism of cofactors and vitamins; 7—Unclassified: metabolism; 8—Energy metabolism; 9—Poorly characterized; 10—Membrane transport; 11—Lipid metabolism; 12—Signal transduction; 13—Metabolism of terpenoids and polyketides; 14—Biosynthesis of other secondary metabolites, 15—Nucleotide metabolism; 16—Xenobiotics biodegradation and metabolism; 17—Translation; 18—Cellular community—prokaryotes; 19—Metabolism of other amino acids; 20—Replication and repair; 21—Folding, sorting and degradation; 22—Glycan biosynthesis and metabolism; 23—Unclassified: signalling and cellular processes; 24—Unclassified: genetic information processing; 25—Cell growth and death; 26—Transport and catabolism; 27—Drug resistance: antimicrobial; 28—Environmental adaptation; 29—Transcription. For further details on this dataset, please see Supplementary Data 1. (**D**) Distribution of the number of reactions per KEGG metabolic pathway across the three organisms. (a) Carbohydrate metabolism; (b) Amino acid metabolism; (c) Metabolism of cofactors and vitamins, (d) Lipid metabolism, (e) Nucleotide metabolism, (f) Metabolism of terpenoids and polyketides, (g) Energy metabolism, (h) Biosynthesis of other secondary metabolites, (i) Xenobiotics biodegradation and metabolism, (j) Metabolism of other amino acids, (k) Glycan biosynthesis and metabolism, (l) Translation, (m) Not included in regular maps, (n) Signal transduction. (**E**) Heatmap with activity index of different KEGG nutrients (y-axis) against the KEGG pathway maps (x-axis). (**F**) Confusion matrix representing a genome-scale model based prediction and the observed growth phenotypes of three organisms. (**B**) was generated using DuctApe^36^, whereas the remaining figures were generated in this study using Python scripts and assembled using Adobe Illustrator 23 https://www.adobe.com/products/illustrator.html.

We analyzed how much genomic content is shared between *S. griseofuscus* DSM40191 and the well-studied model *Streptomyces* strains, *S. coelicolor* A3(2) and *S. venezuelae* ATCC 10712*.* Their genomes were downloaded from NCBI (accession-IDs NC_003888 and NZ_CP029197) and compared to *S. griseofuscus* DSM 40191 by calculating bidirectional best blastp hits between the genes^34^. We found that 3918 genes were shared across all three genomes (core genome), whereas *S. griseofuscus* shared additional 937 and 522 genes with *S. coelicolor* and *S. venezuelae* respectively (Fig. 2A, Supplementary Data 1). The net total of genes present across the three strains (pangenome set) was 13415. In order to understand the biological functions of the shared genes, we annotated all three genomes with the KEGG biological subsystems^35^. For the genome of *S. griseofuscus*, we found 2842 ortholog genes in the KEGG, which are involved in 1498 KEGG reactions. Whereas the genomes of *S. coelicolor* and *S. venezuelae* contained 8152 and 7112 genes, which map to 2900 and 2670 KEGG gene IDs, and 1475 and 1452 KEGG reaction IDs, respectively (Supplementary Data 1). After comparing the KEGG genes and reactions content, we found that the three genomes shared 1488 common KEGG gene IDs and 1280 reactions. Next, we compared the distribution of number of genes and number of reactions belonging to different KEGG pathways across three organisms (Fig. 2C,D). We found that the number of genes involved in subsystems such as membrane transport, signalling and cellular processes were significantly lower in *S. griseofuscus* than in others. However, the number of genes involved in subsystems, such as the metabolism of terpenoids and polyketides, genetic information processing, amino acid metabolism, and xenobiotics biodegradation and metabolism were significantly larger in *S. griseofuscus* (Fig. 2C). Comparison of the number of reactions across different metabolic pathways showed that the three strains shared very similar metabolism. However, the number of reactions involved in pathways, such as amino acid metabolism, was still larger in *S. griseofuscus* (Fig. 2D). Overall, we found that *S. griseofuscus* and *S. coelicolor* had a larger genomic and metabolic content than *S. venezuelae.* Additionally, high genomic and metabolic content was shared between *S. griseofuscus* and *S. coelicolor*. In the later section, we compare the phenotype microarray data of these strains to get experimental understanding of their metabolic growth capabilities.

### Phenotype characterization

#### Comparison of physiological features of *S. griseofuscus*, *S. coelicolor* and *S. venezuelae* using BioLog microarrays

In order to characterize the phenotype of *S. griseofuscus* and its ability to utilize different substrates, we have conducted a multiple parallel cultivation using BioLog microarrays. This technology is not easily applicable for studying actinobacterial strains due to the formation of “clumps'' of mycelia. However, in the case of *S. griseofuscus*, its simple growth characteristics enable such studies. As a direct comparison, we have used the well studied heterologous hosts *S. coelicolor* and *S. venezuelae*. Previously, parallel micro-scale cultivations were used for the characterization of an industrially important *S. lividans* TK24^37^.

We tested a total of 379 substrates, including 190 different carbon sources (PM1 and PM2), 95 nitrogen sources (PM3), 94 phosphate and sulphur sources (PM4). The kinetic growth data from Biolog was analyzed together with the genomes using DuctApe software^36^ that correlated genomic and phenomic data based on the KEGG metabolic pathways. An activity index between 0 to 9 was used to represent the growth on each substrate, where an activity index higher than 3 was used as a cutoff to define growth. We found that 171, 172 and 117 of the 379 substrates were utilized by *S. griseofuscus, S. coelicolor* and *S. venezuelae*, respectively (Fig. 2B, Supplementary Data 2). Comparing their growth, we found that 90 substrates were commonly utilized by all three strains, whereas 14, 19 and 7 substrates were utilized uniquely by *S. griseofuscus, S. coelicolor* and *S. venezuelae*, respectively. Some of the substrates uniquely utilized by *S. griseofuscus* include ethanolamine, 2-aminoethanol, cytidine, thymidine, d-serine and d-threonine. Additionally, we found that *S. griseofuscus* shared a total of 145 common growth substrates with *S. coelicolor*, signalling a high mutual metabolic similarity.

Next, we analyzed the growth on substrates with different nutrient source categories. We observed that a total of 72 carbon sources were utilized by *S. coelicolor*, which was higher than the number of carbon sources used by *S. griseofuscus* (64) and *S. venezuelae* (61). In particular, *S. coelicolor* could utilize more substrates involved in carbohydrate metabolism. Carbon sources uniquely utilized by *S. griseofuscus* include 2-aminoethanol, alpha-keto-valeric acid and D-malic acid. On the contrary, the number of nitrogen sources utilized were higher in *S. griseofuscus* (60) compared to *S. coelicolor* (52) and *S. venezuelae* (49). This could be primarily attributed to the categories of amino acid and other non-defined classes of metabolism. Unique nitrogen sources utilized by *S. griseofuscus* include l-phenylalanine, d-serine and ethanolamine. We found that *S. venezuelae* could only use 6 of the phosphate sources, a number that was substantially lower than in both *S. griseofuscus* (47) and *S. coelicolor* (46). Uniquely utilized phosphate sources by *S. griseofuscus* included 2-aminoethyl phosphonic acid and dithiophosphate. In general, we observe that the capability of *S. griseofuscus* to utilize different nutrient source categories is much higher than that of *S. venezuelae,* and is similar, or even higher than that of *S. coelicolor.* Comparison of the ability of *S. griseofuscus* to grow on different nutrient sources can guide the design of growth media and, thus, leads to optimal growth and metabolite production.

To investigate the connection between these growth activity profiles and the genomic diversity of the strains, a matrix was generated using the *dape* module of DuctApe, where the activity on different nutrients (rows) that are part of different KEGG pathways (columns) is highlighted (Fig. 2E, Supplementary Data 3). For example, the average growth activity indices of all the nitrogen source nutrients belonging to the KEGG pathway of the biosynthesis of amino acids (map:01230) were 6.51, 5.96 and 4.18 for *S. griseofuscus, S. coelicolor* and *S. venezuelae*, respectively. In particular, the thiamine metabolism pathway (map:00730) showed a higher average growth index on nitrogen nutrients in *S. griseofuscus* (7.11) as compared to *S. coelicolor* (5.89) and *S. venezuelae* (5.78). Overall, the growth activity heatmaps of nutrients vs the KEGG pathways were similar in *S. griseofuscus* and *S. coelicolor,* whereas, *S. venezuelae* was found to have lower growth activity across nutrients from different pathways. The higher genomic similarity between *S. grisoefuscus* and *S. coelicolor* that was observed in the previous section further corroborates with this phenomic similarity. In addition to this genome to phenome comparison based on the KEGG pathways, we used genome-scale metabolic models to compare the in-silico predicted growth against observed phenotypes across different substrates (Fig. 2F). We reconstructed draft genome-scale metabolic models for *S. griseofuscus* and *S. venezuelae* based on homology comparison against the genome scale model of *S. coelicolor* (Supplementary Data 4). The models also predicted growth on a larger number of nutrients in the cases of *S. griseofuscus* and *S. coelicolor* as compared to *S. venezuelae* (Supplementary Data 5). Thus, we conclude that *S. griseofuscus* possesses very similar or even superior, metabolic capabilities compared to well-studied *Streptomyces* strains*.*

### Secondary metabolite potential of *Streptomyces griseofuscus*

#### Analysis of the genome using antiSMASH and BiG-SCAPE

In order to estimate the capabilities of the strain to synthesize secondary metabolites, it is important to characterize the BGCs present in the genome. We therefore carried out a genome mining analysis using antiSMASH^3^. We detected 35 regions of BGCs encoding for different types of secondary metabolites on the chromosome. These regions can be split into 53 candidate clusters. The megaplasmid pSGRIFU1 (CP051007) includes one antiSMASH-predicted region with seven candidate clusters. No BGCs were detected on pSGRIFU2 and pSGRIFU3. We observed that the genome of *S. griseofuscus* harbored 4 NRPS, 3 PKSI, 5 PKS-NRPS hybrids, 4 other PKS types, 4 terpenes, 4 RiPPs and 11 other types of BGCs as defined by antiSMASH (Supplementary Table S1). Only few of these BGCs putatively code for known secondary metabolites, such as hopene, geosmin, spore pigment, desferrioxamine B, ectoine and pentamycin. Further, two of the candidate clusters from the plasmid showed similarity to the known BGC encoding for lankamycin and lankacidin C. The remaining 29 BGCs code for unknown and potentially novel secondary metabolites (Supplementary Table S1).

**Figure 3:**
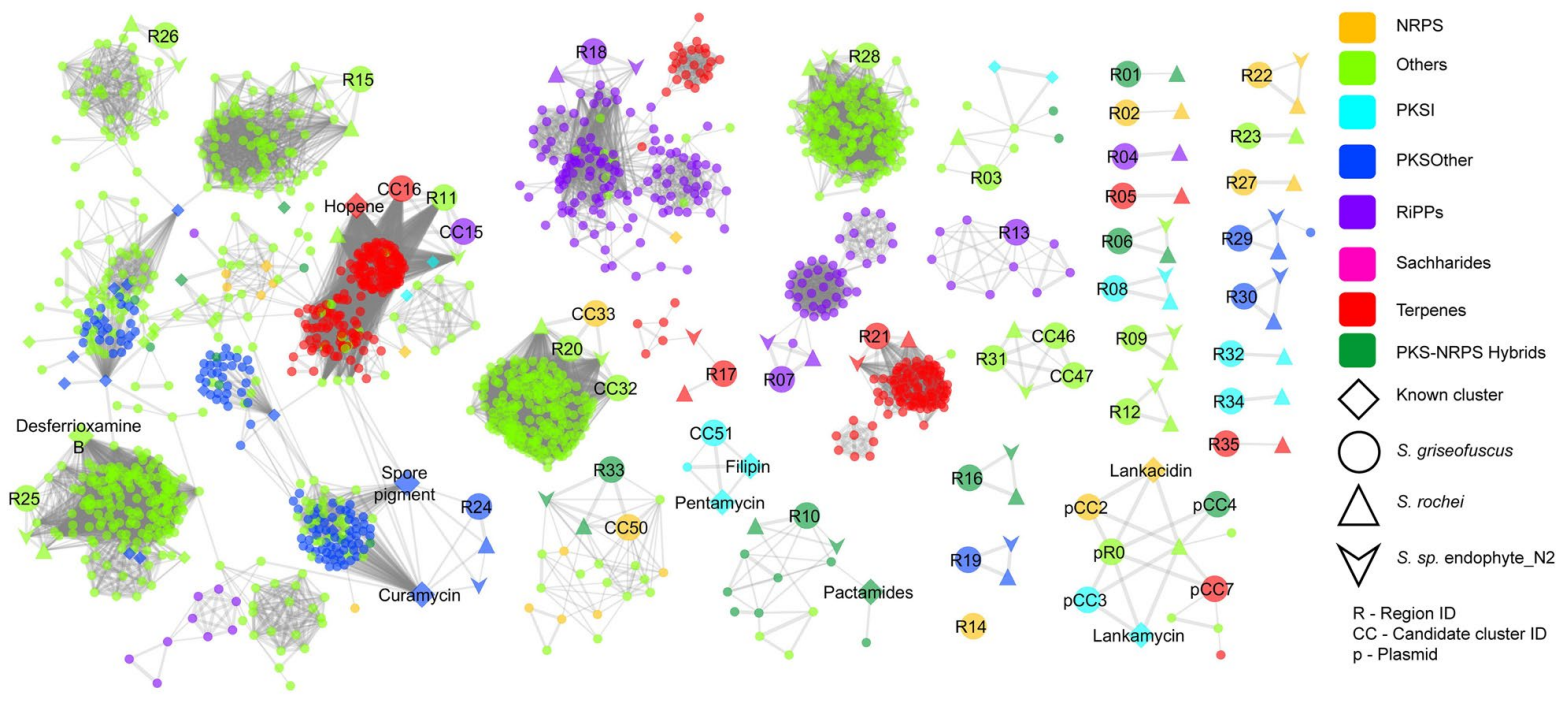
Similarity network of BGCs in *S.griseofuscus* against 212 public genomes and MIBIG database of known BGCs. Comparison of all the regions and few selected candidate clusters against BGCs of known compounds from MIBIG database and 212 public genomes. Different colors denote different types of BGCs as shown in legends. BGCs from *S. griseofuscus, S. rochei* 7434AN4*, Streptomyces. *sp.* endophyte_N2* and MIBIG database are shown with different shapes and sizes. All regions, selected candidate clusters and known BGCs are annotated by text. The similarity network was generated using BiG-SCAPE^38^ and visualised using Cytoscape^39^. Detailed comparison of selected BGCs using CORASON^38^ can be found in Supplementary Fig. S3.

In order to investigate if the BGCs in *S. griseofuscus* are also detected in other *Streptomyces* genomes, we carried out a BGC similarity analysis involving a dataset of 212 publically available complete high-quality *Streptomyces* genomes. In total, 6380 BGCs of different types were detected across this dataset of genomes. We generated a similarity network of 35 regions, 12 manually selected candidate clusters from *S. griseofuscus*, 6380 BGCs from public genomes and 1808 known BGCs from MIBIG database^40^ using BiG-SCAPE^38^. The network with the cutoff of 0.3 *raw_distance* metric was further analyzed using Cytoscape^39^ (Fig. 3). All BGC families that did not include one of the BGCs from *S. griseofuscus* were ignored for the subsequent analyses (Supplementary Data 6)*.* We found that only one BGC (region 14) of the NRPS-like type was a singleton in the network, uniquely observed in *S. griseofuscus*. We observed that 8 of the BGCs were exclusively present in one other genome, namely *S. rochei* 7434AN4. In addition, 9 BGCs are also present in *Streptomyces. sp.* endophyte_N2 (GenBank Accn.: CP028719) in addition to *S. rochei* 7434AN4 (GenBank Accn.: AP018517). This suggests that these 17 BGCs from *S. grieseofuscus* are also rarely observed across streptomycetes*.* Among the BGCs that are relatively common across the dataset, we found that candidate cluster 50 of region 33 had similar BGCs across 18 other *Streptomycetes,* including *S. collinus *Tü 365 (Supplementary Fig. S3), whereas the candidate cluster 51 was similar to the known cluster encoding for pentamycin. Overall, we have established that most of the BGCs (33) were also present in *S. rochei* 7434AN4, indicating two genomes with highly similar content.

This similarity has led us to examine the relation of *S. griseofuscus* to other strains of its species and to *S. rochei* strains. Currently, there are 4 complete assemblies of *S. griseofuscus* and 3 of *S. rochei* genomes available in NCBI database. Among these are the aforementioned *S. rochei* 7434AN4 and type strain *S. rochei* NRRL B-2410. By calculating pairwise Average Nucleotide Identity (ANI) between all genomes^41^, we have identified the ANI between *S. griseofuscus* DSM40191 and *S. rochei* 7434AN4 to be at 99.54%, while the similarity of *S. rochei* 7434AN4 to the type strain *S. rochei* NRRL B-2410 is at 84.03%, highly similar to the one between *S. rochei* NRRL B-2410 and *S. griseofuscus* DSM 40191, 83.95%. This clearly signals that the *S. rochei* 7434AN4 strain was probably misclassified and is indeed a *S. griseofuscus* strain. This may explain the large number of similar BGCs shared between *S. griseofuscus* DSM 40191 and *S. rochei* 7434AN4 and the high levels of similarity between two of the largest plasmids in both strains pSGRIFU1 and pSLA2-L.

#### Characterization of secondary metabolites produced by the *S. griseofuscus*

In the genome mining study, we have identified several known BGC that were studied in other strains. Among these are the lankacidin and lankamycin BGCs, encoded on plasmid pSGRIFU1, previously studied in *S. rochei*^42,43^, as well as the pentamycin BGC33^44^. Due to the good preservation of these BGCs in the genome, we expected to detect the production of lankamycin, lankacidin-related compounds and pentamycin. In addition, it was reported previously, that some strains of *S. griseofuscus* are able to produce azinomycins A and B^45^, acetylcholine esterase inhibitor physostigmine^46^, ϵ-poly-l-lysine^28,29^ and lankacidin C and A^47^. In order to check the production in *S. griseofuscus* DSM 40191, we have performed exploratory cultivations in 5 different liquid media (ISP2, MAM and CDMZ medium^46^, minimal medium (MM)^48^, and medium 65)^49^, that were described in the literature for the production of the compounds mentioned above, and have attempted to identify them in the extracts. Lankacidin A, C, and lankamycin were tentatively identified by HR-MS. The production of pentamycin was identified by HR-MS and confirmed using the pentamycin standard (Supplementary Fig. S4). Physostigmine was not detected in any of the conditions (Supplementary Table S2).

We have noted the production of hydrophobic extracellular vesicles by *S. griseofuscus*, a widely spread, but poorly studied phenomenon among Actinobacteria^50^. It is known that the extracellular vesicles might contain secondary metabolites^51,52^. To study the profile of the extracellular vesicles in *S. griseofuscus*, they were collected and directly injected for LC–MS measurements. Among many compounds, we have tentatively identified lankamycin, that was previously detected in the cultivation extractions.

### Development of genetic engineering methods

Even though the transformation, conjugation and protoplast generation for *S. griseofuscus* was established, including attempts of genetic engineering^31,32,53^, it was never systematically tested with different vectors and engineering methods. While generating a heterologous host strain, it is important to have access to the fastest knockout-leading techniques that lead to the least off-target modifications.

#### Transfer of integrative and replicative GusA-based vectors

As a first step, we tested whether *S. griseofuscus* is compatible with the *gusA* reporter system plasmids^54^: pSETGUS, an integrative phiC31- based plasmid, and pKG1139, a replicative plasmid. Both plasmids were successfully conjugated into *S. griseofuscus* and allowed for visual screening of the exconjugant colonies (Supplementary Fig. S5). To determine the position of the pSETGUS integration site, which is of importance to rationally utilize it for the integration of desirable elements, we randomly picked three independent *S. griseofuscus* pSETGUS colonies and sequenced them using Oxford Nanopore sequencing, similarly to Gren et al.^55^. The exact location of the integration site is at 4,242,328 bp in the HEP81_03793 gene, coding for a putative chromosome condensation protein. The determined *attB* site of *S. griseofuscus* contains the conserved core “TT” sequence^56^.

#### CRISPR-Cas9 mediated gene knockout

CRISPR-Cas9-based molecular tools offer precision and ease in handling in comparison to other techniques. Over the recent years, CRISPR tools have been adapted for use in streptomycetes^57^. As the introduction of double strand breaks can lead to rearrangements and off-target effects in the genome, we validated various CRISPR-Cas9-based engineering methods by targeting genes on the chromosome and on one of the plasmids. For this purpose, we used a pGM1190-based CRISPR-Cas9 plasmid^58^, based on a temperature sensitive replicon that was shown to be functional in *S. griseofuscus*, by using GusA-based pKG1139.

As a first target, we wanted to eliminate plasmid pSGRIFU1 that harbours 4 BGCs, among them the lankacidin, lankamycin, a cryptic polyketide and the carotenoid BGCs. This plasmid has a very high similarity to the plasmid pSLA2-L of *S. rochei,* where these clusters were characterized^43^. A sgRNA was designed to target the DNA primase/helicase-coding region, which is essential for plasmid replication. Three random colonies were selected after the CRISPR procedure and sequenced via Illumina whole genome sequencing. Surprisingly, in all clones, both the targeted pSGRIFU1 and pSGRIFU2 were lost, leaving only plasmid pSGRIFU3 present in the genome. To estimate the amount of changes in the plasmid-cured strains in comparison to the wild type genome, the WGS data was analyzed with breseq, which identified 11 mutations (six SNVs, three insertions, and two deletions) (Fig. 1). One of the colonies was selected for further work and named DEL1.

In parallel, we attempted to knockout chromosomally located BGC region number 33, which encodes a putative pentamycin BGC and an uncharacterized NRPS BGC (Supplementary Fig. S3). The conjugation of the knockout plasmid resulted in less than 10 colonies, 2 of which were selected for Illumina MiSEQ sequencing. It revealed that even though both clones accumulated several mutations, they did not contain the intended mutation (data not shown). Even after the experiment was repeated, we were not able to select knockout-carrying colonies.

In order to verify whether the deletion of pentamycin-NRPS clusters is possible in the plasmid-cured conditions, a knockout plasmid was transferred to DEL1. In contrast to the experiments with the wild type, a large number of exconjugants was received. After the plasmid curing, three of the independently received colonies were sequenced with Illumina NextSeq and one of them was additionally sequenced using Nanopore technology. This clone, further referred to as DEL2, was confirmed to contain a full deletion of the pentamycin-NRPS cluster region and contained a comparatively small amount of mutations (Fig. 1).

In the strain *S. rochei* 7434AN4, which is closely related to *S. griseofuscus*, curing of all three plasmids has been reported to change the topology of the chromosome from linear to circular^43,59^. It is believed that the *tap*-*tpg* gene pair, which encodes for telomere-associated protein and a terminal protein for end patching, located on both pSLA2-L and pSLA2-M plasmids, is responsible for maintaining the linear architecture of the chromosome. Because both the genomes and the associated plasmids in *S. rochei* and *S. griseofuscus* are similar, we investigated if the chromosome of *S. griseofuscus* had circularized during the plasmid curing. We therefore sequenced the strain DEL2 with the Nanopore technology. The assembly graph clearly showed a chromosome with inverted repeat consistent with a linear chromosome. In order to verify the presence of the *tap-tpg* homologues in the genome of *S. griseofuscus*, a BLAST search was performed against each gene pair from pSLA2-L and pSLA2-M. The homologues of *tapR1*-*tpgR1* and *tapRM*-*tpgRM* were found on all three plasmids of *S. griseofuscus*, but not its chromosome (Supplementary Table S4). This could explain the preserved linear topology of the DEL2 chromosome. The removal of the putative pSGRIFU1 and pSGRIFU2 *tap*/*tpg* homologues, may be complemented by the remaining homologous genes present on pSGRIFU3.

Both the DEL1 and DEL2 strains did not show any significant changes in their morphology, growth or sporulation (Supplementary Figs. S1, S2, Supplementary Table S3). In order to verify the influence of genetic manipulations on the metabolites produced by DEL1 and DEL2, parallel cultivations in ISP2 media were made. In comparison to the wild type, strain DEL2 lost the possibility to produce pentamycin, lankacidins and lankamycin, as expected (Supplementary Fig. S4).

In order to test whether *S. griseofuscus* is suitable for the expression of heterologous BGCs, we have expressed the *S. coelicolor* actinorhodin BGC in the wild type and DEL2. As evident from the formation of a dark-blue halo, the wild type and DEL2 strains are both potentially able to produce actinorhodin in heterologous conditions, however further tests are required to unequivocally prove it (Supplementary Fig. S6).

#### CRISPR-cBEST mediated knockouts

The CRISPR-cBEST system^60^ utilizes cytidine deaminase fused to dCas9 and allows for the introduction of STOP-codons by converting CG base pairs to AT. Recently, we have reported the use of this system in *S. griseofuscus*^60^. In order to test the usability of CRISPR-cBEST for engineering of *S. griseofuscus*, the targeted BGCs were selected on the so-called “arm” regions of the chromosome^60^. It is known that the introduction of the DNA double strand breaks by Cas9 might lead to multiple unwanted consequences and is particularly dangerous in the case of the ends of the chromosome^61^. Therefore, these BGC regions are particularly difficult to engineer. In order to verify whether CRISPR-cBEST system would help to omit these limitations, the targets were selected in 4 different BGCs-containing regions, number 4, 30, 31 and 34 on the right and left arms of the chromosome. The pCRISPR-CBE plasmids were constructed according to the protocol^62^, sequenced and transferred to *S. griseofuscus* via conjugation. Correct clones with the STOP-codons in BGC regions 4, 30, 31 and 34 were confirmed via Sanger sequencing of the region of interest^60^. In order to determine the outcomes of each mutation, the morphology, growth and metabolite production was assessed and individually described (Supplementary Figs. S1, S2, Supplementary Table S3). We have grown all of the CRISPR-cBEST generated mutants in ISP2 liquid media and compared their production profiles to the wild type (data is not shown). In the initial tests, we were not able to identify specific metabolites produced from each of these BGCs, possibly because the production conditions for these metabolites were not met, or they are cryptic.

It has been shown that by using the multiplexed CRISPR-cBEST plasmids it is possible to target multiple genes from different BGCs in *S. coelicolor*^60^. Therefore, our next target was to verify such a possibility in *S. griseofuscus*. For this purpose, a multiplex plasmid was constructed, targeting 4 BGCs on the left arm of the chromosome. The sgRNA guides selected earlier were used, yielding plasmid pCRISPR-MCBE-1-2-4-6, targeting BGC region 1 (gene HEP81_00133), BGC region 2 (gene HEP81_00319), BGC region 4 (gene HEP81_00378) and BGC region 6 (gene HEP81_00485). The plasmid was verified via Sanger sequencing and transferred to *S. griseofuscus* via conjugation. Up to 24 exconjugant colonies were tested via PCR. Each of the targeted regions was amplified using a selected set of primers, the fragments were purified and sequenced by Sanger sequencing. As a result of the screening, for each of the targeted regions, at least one successful editing event was detected. We were able to select a colony of *S. griseofuscus* pCRISPR-MCBE-1-2-4-6 with a total of three edited targets (E3I2) (Supplementary Table S5). Strain E3I2 has exhibited signs of sporulation deficiencies and changes in morphology, that might be related to the specific combination of the mutations that were introduced (Supplementary Fig. S1). However, the growth of this strain was clearly not inhibited in liquid cultures (Supplementary Fig. S2, Supplementary Table S3). In addition, the metabolite biosynthesis profile of E3I2 was verified in ISP2 liquid media (data is not shown). We were not able to identify specific metabolites linked to the inactivated BGCs, probably because the conditions for the production of these metabolites were not met or these particular BGCs were not expressed.

One of the significant problems for CRISPR-Cas9-mediated targeting is the unwanted off-target effects. Similarly, such problems exist while using CRISPR-BEST systems. It was shown that while using the CRISPR-BEST for the generation of knockouts in *S. coelicolor*, a relatively small amount of mutations can be observed^60^. However, the influence of the presence of a/the CRISPR-BEST plasmid on the accumulation of the mutations over time during continuous cultivation was never studied.

**Figure 4:**
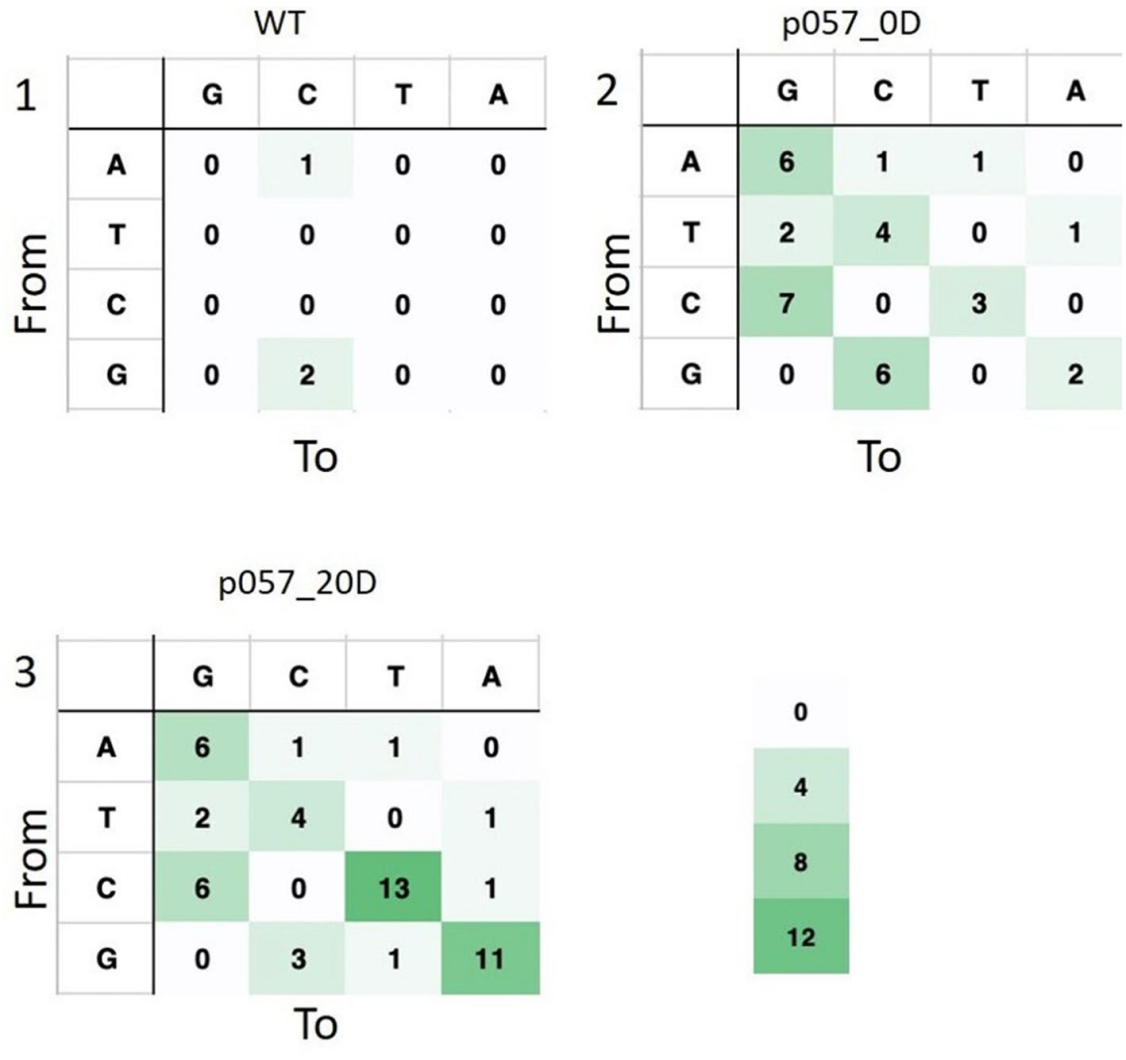
Genome wide off-target evaluation of CRISPR-BEST mediated mutations in the strain preserved and sequenced after the introduction of Stop-codon (p057_0D; 2) in comparison to the same strain that was passed consecutively 20 times in liquid cultures (p057_20D; 3). Mutations, predicted for the WT Illumina dataset (1), used to produce the reference, the level of which can be considered a technical noise. This figure was made using online illustrator draw.io (https://app.diagrams.net/).

In order to study these effects we have performed a long term cultivation experiment with CRISPR-cBEST generated mutant strain *S. griseofuscus* HEP81_06602 (p057). The initial and resulting strains were sequenced using Illumina NextSEQ and compared to the wild type strain, using breseq analysis (Figs. 1, 4). Notably, the introduced Trp221Stop mutation in the putative gene HEP81_06602 (BGC 30) was maintained even after 20 transfers without the antibiotic pressure (Fig. 1).

The breseq analysis of the Illumina-generated reads of the wild type genome had revealed 3 single-base pair mutations, which can be considered a technical baseline. In case of strain p057_0D this number increased to 33 mutations altogether, with a majority of them being C to T exchanges, which can be putatively attributed to nonspecific activity of the CRISPR-BEST cytidine aminase. After 20 consecutive transfers, this number increased to 50 mutations with a majority of them being C to T and A to G exchanges. Both numbers are falling in the range of the previously reported for *S. coelicolor*^60^ and are promising for the engineering of *S. griseofuscus*.

## Discussion

Actinobacterial genomes usually code for 20–30 secondary metabolites^4^. However, under laboratory conditions a limited number of compounds are produced. One strategy to address this challenge is the expression of BGCs in heterologous hosts. Even though there are several hosts available, the current rate of successful expression of BGCs is low^14^. Here, we present the first attempt for diversification of the heterologous strain panel with a potentially easy-to-handle heterologous host, *S. griseofuscus*. The literature-provided information regarding *S. griseofuscus* is comparably dated. Therefore, we have decided to start our work with the wild type strain *S. griseofuscus* DSM 40191 and test its qualities first hand. While exploring its phylogenetic position, we have identified an ANI to *S. rochei* 7434AN4 of 99.54%, while the ANI of *S. rochei* 7434AN4 to the type strain *S. rochei* NRRL B-2410 is only at 84.03%. This indicates that *S. rochei* 7434AN4 may be misclassified, and should rather be included in the *griseofuscus* group. *S. rochei* 7434AN4 is described in detail as a producer of lankamycins, lankacidins and pentamycin^42,44^. However, it was never systematically characterised regarding its genotype, phenotype or genetic engineering abilities. Therefore, detecting the possible similarity between *S. rochei* 7434AN4 and *S. griseofuscus* DSM40191 has been helpful in order to ease the characterisation of *S. griseofuscus* metabolic profile.

The comparison of *S. griseofuscus* genome to genomes of the model streptomycetes *S. coelicolor* and *S. venezuelae* has revealed a close genetic similarity, retained in the ability to process sources of carbon, nitrogen and sulphur. Notably, *S. griseofuscus* is related genetically and phenotypically closer to *S. coelicolor* than to *S. venezuelae*. This enables the use of the methods developed for *S. coelicolor*. In the growth tests, the strain displayed fast, uncomplicated growth in liquid media, compatible with OD measurements, and also an abundant sporulation on solid media. In addition, we demonstrated that *S. griseofuscus* produces the native metabolites lankacidin, lankamycin and pentamycin, as expected from its phylogenetic “proximity” to *S. rochei* 7434AN4. This information was later used in the selection of target BGCs for genetic engineering.

Heterologous host strains have to be frequently manipulated genetically in order to be molded as producers of specific secondary metabolites. Therefore, it is important to have access to a set of efficient genetic engineering techniques. We have tested various methods, such as integrative and replicative vectors, CRISPR-Cas9 based knockouts and CRISPR-cBEST base editing. *S. griseofuscus* is accessible to all these methods, implemented for single or multiplexed knockouts, which generate minimal off-target effects. Even though CRISPR-based systems were shown to be applicable in Actinobacteria, their use is still largely limited to model strains and typically requires further adjustments^63^. It is, therefore, highly beneficial that *S. griseofuscus* does not require any of such additional efforts.

Our first target for the genome reduction of *S. griseofuscus* has been the curing of the largest plasmid pSGRIFU1, highly similar to pSLA2-L of *S. rochei*^44^. It carries 4 BGCs, among these the lankacidin and lankamycin BGCs, products which were detected in the wild type supernatant. As expected^42^, CRISPR-Cas9 mediated curation of pSGRIFU1 led to the disappearance of these metabolites in the supernatants of the mutant strain DEL1. To generate strain DEL1, we targeted the DNA primase/helicase-coding region of the pSGRIFU1 plasmid, which also caused the curing of plasmid pSGRIFU2. This could be explained by the presence of several putative off target sites in pSGRIFU2. Curation of pSGRIFU2 was unexpected, but a positive outcome for the genome minimization and did not lead to chromosome cyclisation of DEL1, as it was shown for *S. rochei*^59^. We believe that the homologues of genes *tap*/*tpg*, that were detected on plasmid pSGRIFU3, are needed to sustain the architecture of the chromosome.

Furthermore, we have successfully removed pentamycin and the NRPS BGC from the genome of DEL1, generating strain DEL2, deficient in pentamycin production (Supplementary Fig. S5). DEL2 can be considered as the first genome reduced strain of *S. griseofuscus* with 5.19% of its genome removed without any observable negative effects. In addition, the heterologous expression of actinorhodin BGC in strain DEL2 has resulted in formation of a blue halo, possibly indicating the production of actinorhodin (Supplementary Fig. S6). Therefore, we believe that strain DEL2 can be used for further genome minimization and stepwise improvements towards its development as a heterologous production host.

## Conclusions

For the last decades, *S. griseofuscus* has remained largely unexplored. In this paper, a detailed characterization of *S. griseofuscus* DSM 40191 is presented, including studies of its genotype, phenotype and metabolic capabilities. We believe that the results, described in this manuscript, clearly demonstrate the high potential of *S. griseofuscus* strain as to its further development as a heterologous host strain. We plan to continue our work on the genome reduction of *S. griseofuscus* DEL2, using step-by-step removal of BGCs with the simultaneous characterisation of the mutants growth and metabolite production. In the next phase of development, we plan to adapt the resulting strain to expression of different types of BGCs, by introducing additional integration sites and by modifying the primary metabolism to suit the precursor needs.

## Materials and methods

### Strains, used or constructed in this study

Strains *S. griseofuscus* DSM 40191 and *S. venezuelae* DSM 40230 were received from DSMZ strain collection as freeze-dried pellets. Strain *S. coelicolor* M145 was received from Y. Tong (NBC group, DTU, Denmark). *E. coli* Top10 and Mach1 (both from Thermo Fisher Scientific) were used as general cloning hosts in this study. Other strains that were used in this study are described in Supplementary Table S5.

### Growth and cultivation conditions

#### Growth and sporulation on different media

To systematically study a phenotype of *S. griseofuscus*, we tested several solid media, detailed below (Supplementary Fig. S1, Supplementary Table S3). To confirm the disperse growth in liquid media, we have built a growth curve based on optical density at 600 nm (OD_600_) in liquid ISP2 (Supplementary Fig. S2, Supplementary Table S1). All of the *Streptomyces* strains were cultivated using 3 different solid media: MS^48^, ISP2 and ISP4 [International Streptomyces Project medium 2 and 4; premixed Difco ISP2 dehydrated medium (Fisher Scientific, #DF0770-17-9) and premixed Difco ISP4 dehydrated medium (Fisher Scientific, #DF0772-17-7)]. DSMZ medium 65 (GYM *Streptomyces* medium) is the medium recommended by the German Collection of Microorganisms and Cell Cultures (DSMZ) for cultivation of *S. griseofuscus* DSM 40191 and prepared as described by DSMZ^49^. CDMZ, a chemically defined media for the detection of physostigmine, was prepared as described in Ref.^46^. Otherwise, *Streptomyces* minimal media^48^, MAM, molicidin A medium, and version of ISP2 without addition of agar, were used for liquid media cultivations. For cultivations in liquid medium, 250 ml shake flasks were used in combination with approximately 30 3 mm glass beads, incubated at 30 °C and 200 rpm. For the cultivations in liquid media, a two stage cultivation principle was used. Firstly, pre-cultures were directly inoculated with spore suspensions that were collected from fully grown MS plates, and incubated overnight. The OD600 was measured for each pre-culture and re-inoculated in the main culture up to the final OD600 of 0.1. The OD600 measurements were carried out in the 1.5 ml disposable polystyrene spectrophotometer cuvettes. Before each measurement, the cuvettes were thoroughly mixed using a vortex-mixer and each cuvette was measured three times. Therefore, each presented data point corresponds to a total of nine measurements. The maximum specific growth rate µ_max_ was determined by plotting the natural logarithm of all OD_600_ values against the cultivation time. Further on, a linear fit for data points corresponding to the exponential phase was calculated and the slope of that fit corresponds to µ_max_. Using µ_max_, the minimal time needed for the OD_600_ value to double (td) was calculated as described in Ref.^64^.

### Cloning, primers and constructed plasmids

Spacer sequences, primers, constructed and used plasmids are listed in Supplementary Table S5. All spacer sequences were selected with the help of CRISPy-web^65,66^.

The procedure of ssDNA oligo bridging was used for the integration of spacers into CRISPR plasmids. The CRISPR plasmid of interest was digested with NcoI at 37 °C for 30 min and dephosphorylated using FastAP at 37 °C for another 30 min. The reaction was inactivated by incubation at 65 °C for 10 min. The 20 nt spacers were ordered as oligos from IDT with 20 nt overlaps to the backbone on both sides (Supplementary Table S5). After dilution to 100 µM, the oligos were diluted to a concentration of 0.2 µM using 1 × NEBuffer 2. A 10 µl reaction mix was prepared containing 30 ng of the linearized backbone, 5 µl of the 0.2 µM oligo, and ddH_2_O to 10 µl. 10 µl of NEBuilder HiFi DNA Assembly Master Mix were added and the reaction was incubated for 1 h at 50 °C. Up to 5 µl were transformed into electrocompetent *E. coli* Mach1. Positive colonies were identified by colony PCR and running the samples on a 2% agarose gel. Putative positive colonies were further analyzed by the in-house Sanger sequencing. The construction of CRISPR-Cas9 plasmids and their transfer was carried out as described in Ref.^62^. All *E. coli-Streptomyces* conjugation experiments were conducted according to the modified protocol from Ref.^48^, from which the heat shock step was completely omitted. The MS media with addition of magnesium chloride solution^48^ was used for plating of conjugation mixes.

### Genome comparison of *S. griseofuscus, S. coelicolor and S. venezuelae*

To compare genetic features of *S. griseofuscus* with other well studied *Streptomyces* strains, we downloaded the genomes of *S. coelicolor* A(3)2 (NCBI accession.: NC_003888) and *S. venezuelae* ATCC 10712 (NCBI accession: NZ_CP029197). All three genomes were annotated using the KEGG annotation server that describes the biological subsystem of each gene^35^. The number of genes in different biological subsystems were counted for each genome. Similarly, to compare the metabolic properties, the number of unique KEGG reactions were counted for different metabolic pathways across the three genomes. The genomic and phenotypic microarray data was analyzed together using the *dape* module of the DuctApe software^36^ that is used to correlate genomic data with phenomic data for multiple strains. As part of this software, bidirectional best blast hits (E-value threshold − 1e^−10)^ were calculated to generate the pangenome of the three strains. Based on this pangenome, the number of shared genes, unique KEGG gene IDs, and unique KEGG reaction IDs across the three genomes were calculated.

### BioLog phenotypic microarrays

Phenotype MicroArrays were ordered from BioLog, which in addition had provided customized protocol for the cell inoculation and measurements. In this study, testing plates PM 1 to 4 were used, out of which PM1 and 2 contain Carbon sources, PM2—Nitrogen sources, PM4—Phosphorus and Sulphur sources. All the measurements for all strains were performed in three technical and two biological replicates. *S. coelicolor* M145, *S. venezuelae* DSM 40230 and *S. griseofuscus* DSM 40191 were all grown on MS plates for 6 days until clear sporulation signs appeared. The spores were collected in sterile water and diluted to 80% of cell density using a BioLog-supplied turbidimeter (catalogue number: 3531). BioLog redox dye mix G was used for all measurements. The recipes for minimal media and other supplementing resources were received from BioLog. The cells were mixed with the prepared media and inoculated in the PM plates using multichannel pipettes and immediately loaded into the OmniLog instrument for measurements (catalogue number: 91171). The kinetic data for all testing plates PM1 to PM4 generated by BioLog were further analyzed using DuctApe software^36^ along with the genomic data. Activity index between 0 to 9 was assigned for each nutrient source to represent the growth activity on each nutrient source. Average activity index was further calculated for the two replicas that were used to generate phenotype data. The average activity index across 379 nutrient sources across 4 PM plates was visualized using activity index rings for the three different strains (Supplementary Data 2). Sources with differential growth activities were further analyzed. To compare the growth activity with genomic features, a matrix with activity on different KEGG nutrients (rows) against KEGG pathways with the nutrient (columns) was calculated. These matrices were compared using heatmaps where rows and columns are ordered as per *S. griseofuscus* growth activity index and number of reactions per KEGG pathway respectively (Supplementary Data 3).

We used Python‐based genome-scale model reconstruction tool ‘Genome-scale Modelling with Secondary Metabolism’ (GMSM) that implements bidirectional blastp hits-based homology modelling^67^ to automatically reconstruct models for both *S. venezuelae* and *S. griseofuscus*. We used a model of *S. coelicolor* A3(2), iMK120848, as a template for the homology modelling. The in-silico growth predictions were made using flux balance analysis on different simulated mediums using COBRApy. Confusion matrix was created from predicted and observed growth phenotypes to compare the model prediction against Biolog data.

### DNA isolation, sequencing and assembly

As described in Ref.^33^, WGS libraries were constructed using the KAPA (St. Louis, Missouri, USA) HYPRplus kit and sequenced on an Illumina MiSeq machine with a 2 × 150 nt sequencing kit, except for the library for strain DEL2, which was sequenced on a Illumina NextSeq 500. The Illumina data was adaptertrimmed using Adapterremoval2 (v. 2.2.2)^68^ with the switches --trimns --trimqualities. As described in Ref.^33^, the genome of *S. griseofuscus* was assembled using PacBio data with the assembly program Flye (v. Flye 2.4.1-geb89c9e)^69^ with the switches --genome-size 8 m --iterations 5 for five consecutive rounds of polishing using the PacBio data. The assembly was then polished with the Illumina data using the polishing module of Unicycler (v. 0.4.8-beta)^70^. We manually added the inverted repeats to both ends of the chromosome, repeated the illumina polishing with unicycler-polish, and used Minimap2 (v. 2.16-r922)^71^ and Bowtie2 (v. 2.3.5)^72^ to map pacbio and illumina reads to the manually curated genome assembly. We used artemis genome viewer (v 0.18.0.2)^73^ to visualize the mappings. BUSCO (v. 4.0.5)^74^ was used for estimating the quality of the genome assembly and Bandage (v. 0.8.1)^75^ was used to view and evaluate the assembly graph. The assembled genome was gene annotated using Prodigal (v.2.6.3)^76^ and the identified genes were functionally annotated using Prokka (v. 1.14.0)^77^ with the PFAM-A (v. 32.0)^78^ database and six publically available manually annotated actinobacterial genomes of high quality using the prokka --proteins switch. These databases were used in addition to the default databases, not instead of them. RNAmmer^79^ was used for rRNA gene prediction and Aragorn was used for tRNA prediction^80^. Nanopore data from the strain DEL2 was obtained to check for circularization of the genome. The DNA of the DEL2 strain was extracted as described above, and a nanopore library was constructed with the rapid (SQK-RBK004) kit from Oxford Nanopore Technologies (Oxford, United Kingdom) and the barcode RB05. The data was generated on a MinION machine with a 9.4.1 flowcell. The raw data was demultiplexed using Deepbinner (v. 0.2.0)^81^ and basecalled using Guppy (v. 3.2.2 + 9fe0a78, Oxford Nanopore Technologies, Oxford, United Kingdom), before the technical sequence was removed using Porechop (v. 0.2.4; https://github.com/rrwick/Porechop). The Nanopore data was assembled using flye assembler as described above for PacBio data.

### Comparison of BGCs of *S. griseofuscus* with BGCs detected across *Streptomyces *sp*.*

Genome sequences with complete assembly annotation of the *Streptomycetaceae* group are collected from the PATRIC database^82^. Among 214 complete genome sequences, one was annotated of poor quality owing to its low fine consistency score (PATRIC ID: 2588708.3) and one other genome had 22 contigs (PATRIC ID: 1969.5). The remaining 212 high quality genomes are processed with antiSMASH v5.1 to detect 6380 BGCs in total. The antiSMASH predicted 34 BGCs on chromosome and one BGC on plasmid of *S. griseofuscus*. Some of these BGCs were further split into candidate clusters manually. We used BiG-SCAPE software^38^ to generate similarity network of all 6380 BGCs detected in 212 public genomes, 1808 known BGCs from MIBIG database v1.3, 35 BGCs from *S. griseofuscus* genomes and manually selected 14 candidate clusters. The cutoffs of 0.3, 0.5 and 0.7 were used on raw_index similarity metric of BiG-SCAPE analysis. The similarity network was visualized using Cytoscape v3.7^39^. Some of the candidate clusters of interest (e.g. candidate cluster number 5) were analyzed using the CORASON feature of BiG-SCAPE to generate gene cluster alignments based on similarity.

### Extractions and evaluation of metabolite production

For targeted and untargeted metabolomics analysis, 50 ml liquid cultures with the medium of interest (ISP2 and CDMS) were prepared and cultivated in 250 ml shake flasks for 5 days. After 5 days, the cultures were harvested and centrifuged for 20 min at 10,000×*g*. The supernatants were collected in 250 ml glass bottles and extracted using equal volumes (1:1) of ethyl acetate (liquid–liquid extraction). The organic phase was separated from the aqueous phase using a 500 ml separatory funnel, after shortly shaking, and collected in a 250 ml glass bottle. The process was repeated 3 times using fresh solvent. The extracts of each strain were combined and evaporated using a Büchi Rotavapor R-300 in combination with a Büchi Heating Bath B-300 Base, a Büchi Interface I-300, a Büchi Vacuum Pump V-300, and a Julaba Recirculating Cooler FL601. The temperature of the water bath was set to 38 °C, the rotation speed to 140 rpm, and the pressure to 150 mbar, which was gradually decreased to 50 mbar to achieve complete dryness. Next, the analytes were dissolved in 1 ml of 50% v/v methanol and transferred to 2.0 ml Eppendorf tubes. The samples were centrifuged for 10 min in 12,000 CFU and the supernatants were transferred in new eppendorf tubes. The samples were evaporated again using an Eppendorf Concentrator Plus Speedvac without heating, dissolved in 100 µl of 50% v/v methanol and centrifuged for 10 min in 12,000 CFU. The supernatants were transferred to autosampler vials with inserts, and analyzed using a LC–MS system. The analysis was performed using a Thermo Dionex Ultimate 3000 UHPLC system with a diode array detector (DAD) interfaced with an Orbitrap Fusion Tribid mass spectrometer (Thermo Scientific, San Jose, USA), using an EASY-IC ESI source. Separation conditions were as follows: Column, Agilent Zorbax Eclipse Plus C18, 100 × 2.1 mm i.d., 1.8 µm particles. The mobile phase used was (A) purified water with 0.1% formic acid and (B) acetonitrile with 0.1% formic acid. The flow rate was set to 0.35 ml/min and the column temperature was set to 35 °C, while the injection volume was set to 1 µl. The following gradient was used: 0–0.5 min. 5% B, increasing to 100% B at 13 min., and holding until 15 min., returning to 5% B at 15.1 min., and equilibrating for 1.9 min., for a total run time of 17 min. Full-scan mass spectrometric detection was performed in positive and negative ESI mode with the following parameters: source voltage, 3500 V (positive mode) and 2700 V (negative mode); sheath gas flow rate (N2), 50 arbitrary units; auxiliary gas, 10 arbitrary units; sweep gas, 1 arbitrary unit; Ion transfer tube temperature, 325 °C; Vaporizer temperature, 350 °C. MS full-scan analysis was performed using the orbitrap with the following settings: Orbitrap resolution, 120,000; scan range 100–1000 Da; RF lens, 50%. Before analysis, the MS was calibrated using ESI Positive Ion Calibration Solution (P/N 88323) and ESI Negative on Calibration Solution (P/N 88324, Thermo Scientific, San Jose, USA). Fragmentation data for compound annotation were obtained using data-dependent MS/MS analysis by selecting the top four most intense ions per cycle. Dynamic exclusion was used to exclude ions for 3 s after two measurements within 4 s. Fragmentation was performed using an assisted fragmentation HCD 15, 30, 45, and 60% at a resolution of 30,000 with an AGC target of 1 × 10^5^ and a maximum injection time of 64 ms. Data analysis was performed using Thermo Scientific Compound Discoverer 3.0.0.294. Using the software, data from the LC–MS analysis were aligned, and compound annotation was performed by matching against molecular formulas from StreptomeDB2 and AntiBase, as well as fragmentation spectra from mzCloud. In the case of pentamycin detection, a pure pentamycin standard was used, diluted in 10% v/v methanol to 10^–3^ mg/ml concentration. In regards to the other compounds, no standards were measured, due to their absence in the market, so their detection is putative.

## Supplementary Information

Supplementary Information.

## Data Availability

The GenBank accession numbers for *S. griseofuscus* genome are CP051006 (chromosome), CP051007 (pSGRIFU1), CP051008 (pSGRIFU2), and CP051009 (pSGRIFU3). All Supplementary Data files 1–6, including Excel tables and XML format model files, are available at 10.11583/DTU.13032803.
