# Supplementary material for: Characterization and engineering of Streptomyces griseofuscus DSM 40191 as a potential host for heterologous expression of biosynthetic gene clusters
Source: Sci Rep. 2021 Sep 15;11:18301. doi: 10.1038/s41598-021-97571-2 (PMC8443760; doi:10.1038/s41598-021-97571-2)
Supplement: Supplementary file 1 — Supplementary Information. [file 41598_2021_97571_MOESM1_ESM.pdf]

Supplementary Information

Characterization and Engineering of *Streptomyces griseofuscus* DSM 40191 as a Potential Host for Heterologous Expression of Biosynthetic Gene Clusters

Tetiana Gren[a], Christopher M. Whitford[a], Omkar S. Mohite[a], Tue S. Jørgensen[a], Eftychia E. Kontou[a], Julie B. Nielsen[a], Sang Yup Lee[a,b], Tilmann Weber[a#]

[a]The Novo Nordisk Foundation Center for Biosustainability, Technical University of Denmark, Denmark

[b]Metabolic and Biomolecular Engineering National Research Laboratory, Department of Chemical and Biomolecular Engineering, Center for Systems and Synthetic Biotechnology, Institute for the BioCentury, Korea Advanced Institute of Science and Technology, Republic of Korea

[#] correspondence address:
Prof. Dr. Tilmann Weber
The Novo Nordisk Foundation Center for Biosustainability
Technical University of Denmark
Kemitorvet, bygning 220
2800 Kgs. Lyngby
Denmark
Email: tiwe@biosustain.dtu.dk
Phone: +45 24 89 61 32

Supplementary Information 1

Figures and Tables 3

Figure S1 3

Figure S2 4

Figure S3 5

Figure S4 6

Figure S5 7

Figure S6 8

Table S1 9

Table S2 11

Table S3 12

Table S4 13

Table S5 14

Figures and Tables

Figure S1

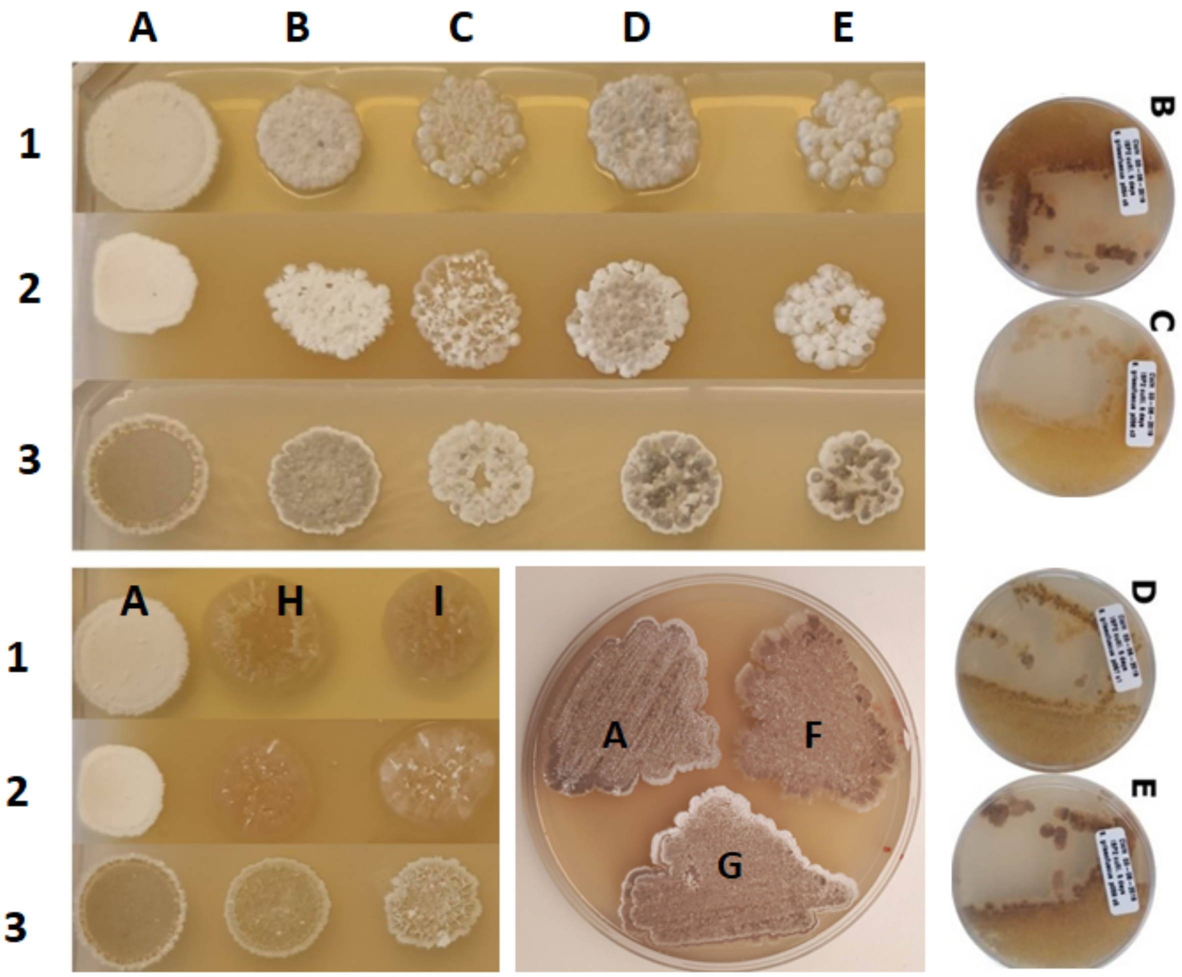

Figure S1. Phenotype of *S. griseofuscus* strains on solid media. 1 - ISP2 media; 2 - MS media; 3 - ISP4 media. A- wild type; B - *S. griseofuscus* IppsD_1 (p054); C - *S. griseofuscus* ItycC_2 (p056); D -*S. griseofuscus* HEP81_06602 (p057); E - *S. griseofuscus* IspkC (p059); F - *S. griseofuscus* DEL1; G - *S. griseofuscus* DEL2; I - *S. griseofuscus* E3I2

Figure S2

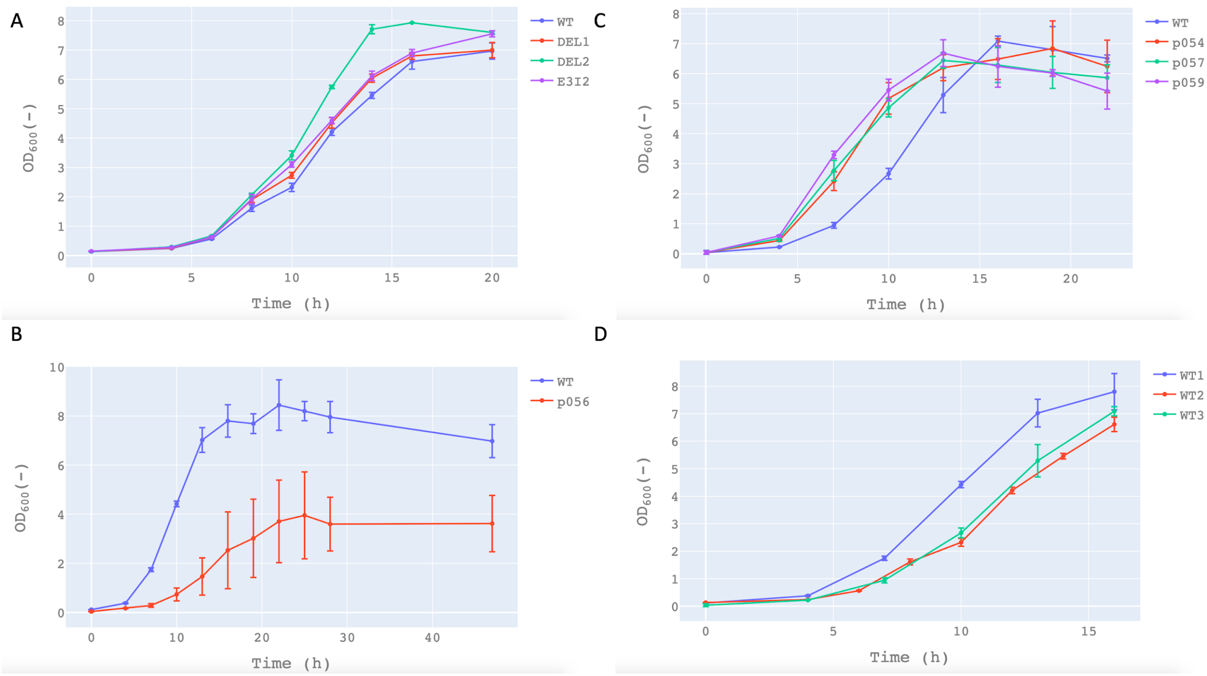

Figure S2. OD600 measurements of *S. griseofuscus*-derived strains, while grown in ISP2 liquid media. A - characterization of DEL1, DEL2, E3I2 strains in comparison to the wild type; B - characterization of slow growing strain p056 in comparison to the wild type; C - characterization of *S. griseofuscus* IppsD_1 (p054), *S. griseofuscus* HEP81_06602 (p057) and *S. griseofuscus* IspkC (p059) strains in comparison to the wild type; D - comparison of all 3 independant cultivations of wild type strains.

Figure S3

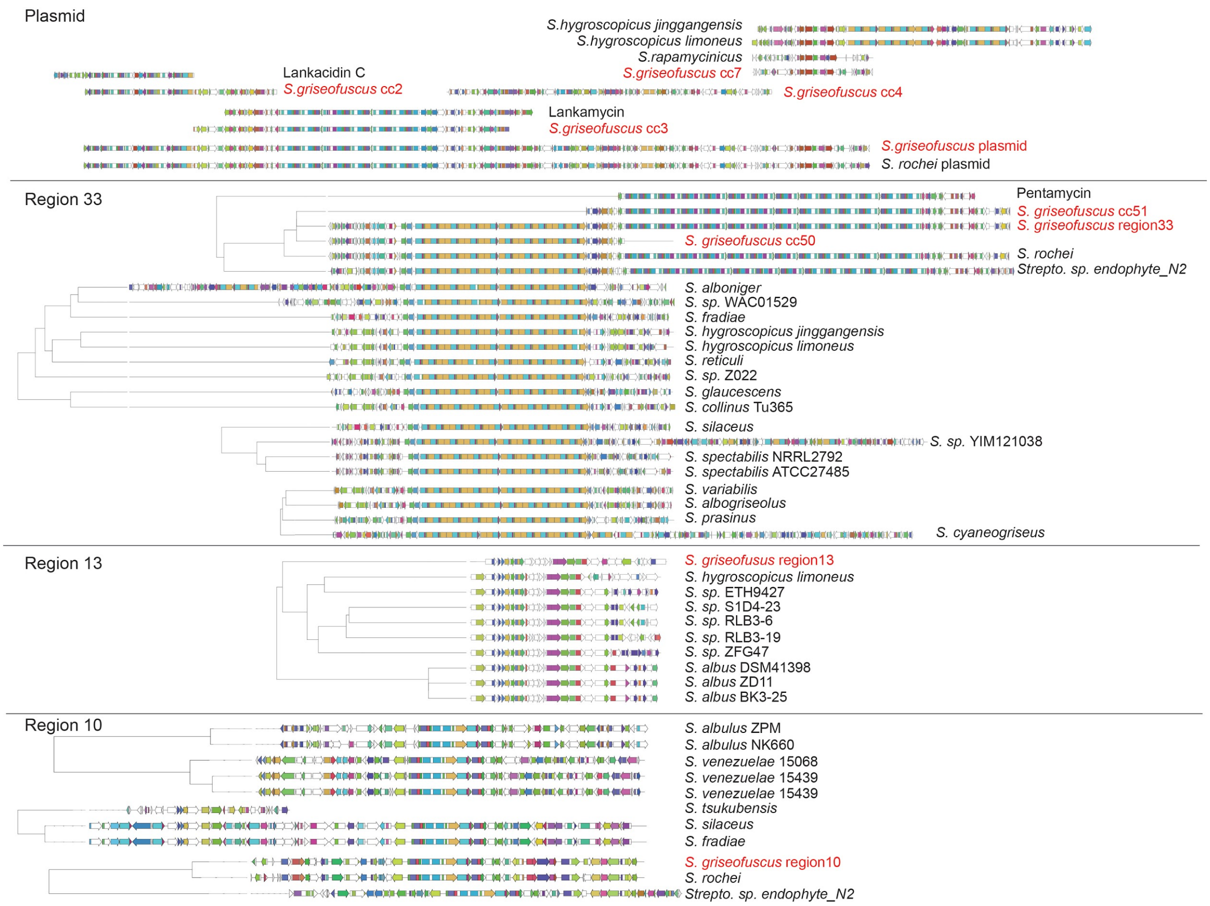

Figure S3. Alignment of selected families of BGCs. Here selected regions and candidate clusters of *S. griseofuscus* are aligned against similar BGCs detected across the dataset. Note: pentamycin BGC was manually added in the above analysis as default BiGSCAPE uses an older MIBIG version, lacking pentamycin BGC.

Figure S4

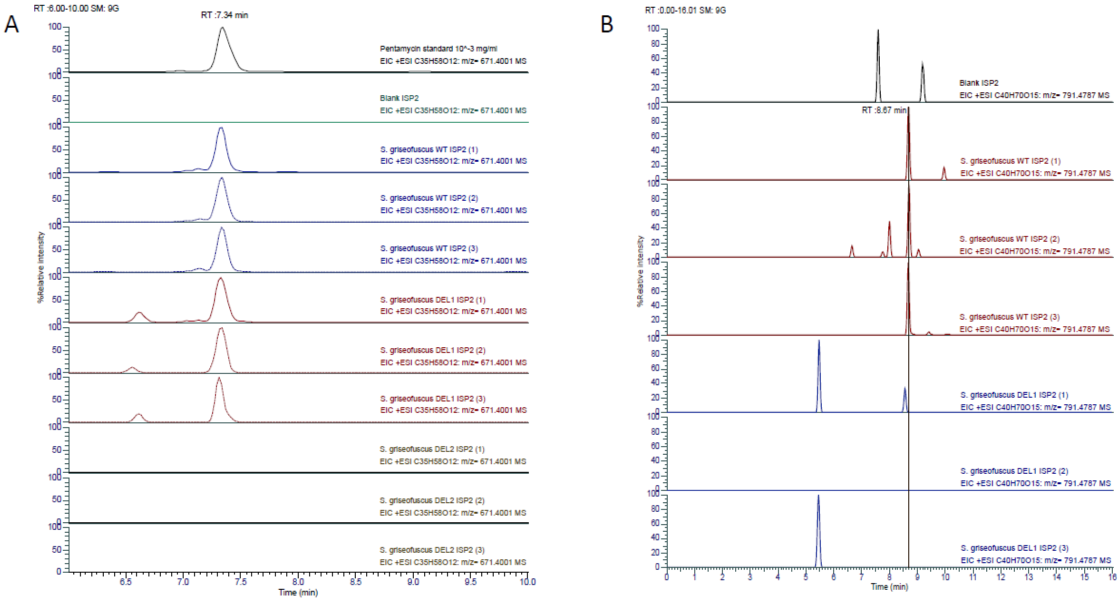

Figure S4. Results of metabolite production analysis of cultivations and extractions of wild type, DEL1 and DEL2 strains, grown in liquid ISP2 media. A) extracted ion chromatograms of pentamycin production of strains WT, DEL1 and DEL2, cultivated in ISP2 media in comparison to the extraction of pure ISP2 media and pentamycin standard; B) the extracted EICs for kujimycin A are plotted for comparative metabolomics between blank ISP2 medium, three replicates of WT and three replicates of DEL1 in ISP2. The peak at 8.70 min is a putative match for the molecule and its clearly absent in case of DEL1 strain.

Figure S5

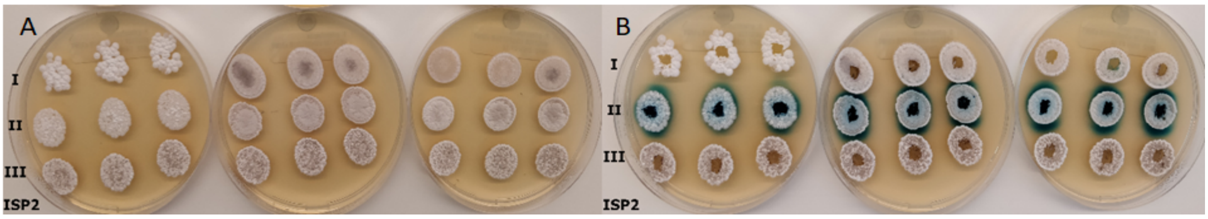

Fig. S5. A and B - three independent colonies of *S. griseofuscus* pKG1139 (I), *S. griseofuscus* pSETGUS (II) and wild type (III), grown on ISP2 solid media after the 24 hours incubation at 40°C to verify possibility of plasmid curing, A- colonies before the addition of X-Gluc; B - colonies after the addition of X-Gluc.

Figure S6

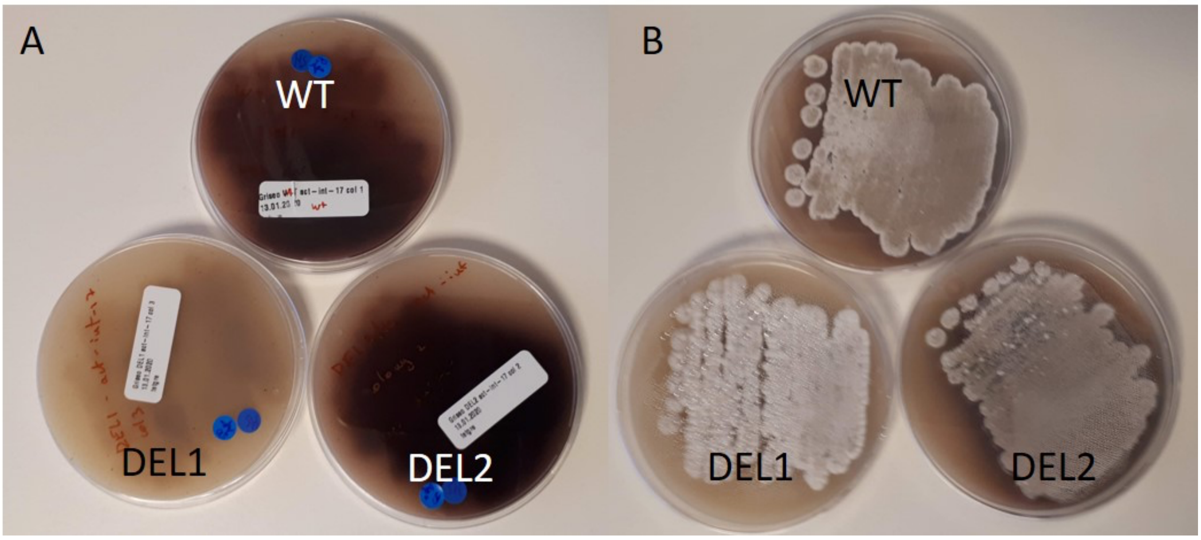

Figure S6. Strains *S. griseofuscus* wild type, DEL1 and DEL2, that contain integrative plasmid construct, carrying complete actinorhodin gene cluster, cloned from *S. coelicolor* M145. All strains were grown on MS media, supplemented with apramycin for 6 days before the photo was taken. Wild type and DEL2 strain, carrying actinorhodin gene cluster, produce dark blue halo, while no such halo is observed in case of strain DEL1

Table S1

Table S1. antiSMASH predicted BGCs in the genome of *S. griseofuscus* with information on selected candidate clusters.

Region number	Protoclusters	Cluster type	Cluster size	From- To	Known/ Unknown cluster
1	CC1	NRPS/T1PKS	64,650	112,225 - 176,874	Unknown
2	CC2	NRPS-like	42,325	310,053 - 352,377	Unknown
3	CC3	Siderophore	10,344	371,242 - 381,585	Unknown
4	CC4, CC5	Other/Lantipeptide	39,420	392,231 - 431,650	Unknown
5	CC6	Terpene	19,939	472,998 - 492,936	Unknown
6	CC7	NRPS/T1PKS	83,447	493,026 - 576,472	Unknown
7	CC8	Bacteriocin	7,905	748,506 - 756,410	Unknown
8	CC9	T1PKS	41,295	783,802 - 825,096	Unknown
9	CC10	NRPS/Lanthipeptide	69,518	887,334 - 956,851	Unknown
10	CC11, CC12, CC13	NRPS/T1PKS/transAT-PKS-like	49,506	1,071,986 - 1,121,491	Unknown
11	CC14, CC15, CC16	LAP/Thiopeptide/Terpene	52,196	1,283,460 - 1,335,655	Unknown
	CC15	LAP/Thiopeptide	45,062	1,283,460 - 1,328,521	Unknown
	CC16	Terpene	25,786	1,309,870 - 1,335,655	Hopene
12	CC17	NRPS/Betalactone	88,583	1,421,074 - 1,509,656	Unknown
13	CC18	Lanthipeptide	25,183	1,676,415 - 1,701,597	Unknown
14	CC19, CC20, CC21	NRPS-like	43,318	1,808,661 - 1,851,978	Unknown
15	CC22, CC23	NRPS/Siderophore	71,151	1,916,492 - 1,987,642	Unknown
16	CC24	NRPS/T1PKS	51,706	1,992,803 - 2,044,508	Unknown
17	CC25	Terpene	20,640	2,150,766 - 2,171,405	Unknown
18	CC26	Bacteriocin	11,326	2,183,111 - 2,194,436	Unknown
19	CC27, CC28, CC29, CC30	PKS-like/T1PKS/Other	57,557	2,320,695 - 2,378,251	Unknown
20	CC31, CC32, CC33	NRPS/Siderophore	47,491	2,550,673 - 2,598,163	Unknown
	CC32	Siderophore	10,357	2,550,673 - 2,561,029	Unknown
	CC33	NRPS	43,389	2,554,775 - 2,598,163	Unknown

21	CC34	Terpene	19,893	3,159,315 - 3,179,207	Unknown
22	CC35	NRPS	54,105	3,767,999 - 3,822,103	Unknown
23	CC36	Butyrolactone	10,933	4,004,072 - 4,015,004	Unknown
24	CC37	T2PKS	72,512	4,642,100 - 4,714,611	Spore pigment
25	CC38	Siderophore	11,770	5,480,347 - 5,492,116	Desferrioxamine E
26	CC39	Melanin	10,597	5,564,462 - 5,575,058	Melanin
27	CC40	NRPS-like	42,938	5,828,280 - 5,871,217	Unknown
28	CC41	Ectoine	10,090	6,446,392 - 6,456,481	Ectoine
29	CC42, CC43, CC44	T1PKS/hglE-KS	51,804	7,049,867 - 7,101,670	Unknown
30	CC45	T3PKS	41,065	7,167,442 - 7,208,506	Unknown
31	CC46, CC47	NRPS/Ectoine	114,646	7,212,385 - 7,327,030	Unknown
	CC46	NRPS	114,646	7,212,385 - 7,327,030	Unknown
	CC47	Ectoine	10,378	7,314,117 – 7,324,494	Unknown
32	CC48	T1PKS	46,595	7,342,702 - 7,389,296	Unknown
33	CC49, CC50, CC51	NRPS/T1PKS	155,935	8,241,194 - 8,397,128	Unknown
	CC50	NRPS	78,929	8,241,194 – 8,320,122	Unknown
	CC51	T1PKS	96,884	8,300,245 - 8397128	Pentamycin
34	CC52	T1PKS	47,647	8,471,303 - 8,518,949	Unknown
35	CC53	Terpene	25,692	8,603,150 - 8,628,841	Careotenoid
Plasmid	CC1 to CC7	NRPS/T1PKS/T2PKS/butyrolactone/terpene	173,752	10,919 - 184,670	Unknown
	CC2	NRPS	42,435	10,919 – 53,353	Lankacidin
	CC3	T1PKS	69,767	35,247 – 105,013	Lankamycin
	CC4	T1PKS/T2PKS/NRPS/butyrolactone	72,513	90,336 – 162,848	Unknown
	CC7	Terpene	25,688	158,983 – 184,670	Unknown

Table S2

Table S2. Compounds detected in ethyl acetate extractions of supernatants of S. griseofuscus wild type cultivations in different liquid media. RT - retention time, MM - minimal medium, M65 - medium 65. Detection of pentamycin was confirmed via comparison with a pure pentamycin standard.

**Compound**	**RT (min)**	**MM**	**CDMZ**	**ISP2**	**MAM**	**M65**
Azinomycin A	-	-	-	-	-	-
Azinomycin B	-	-	-	-	-	-
Lankacidin C	6.07	Detected	Detected	Detected	-	Detected
Lankacidin A	7.93	Detected	Detected	-	-	-
Lankamycin	9.47	-	Detected	-	-	-
Pentamycin	7.43	-	Detected	-	-	-
Physostigmine	-	-	-	-	-	-

Table S3

Table S3. OD600 measurements of *S. griseofuscus*-derived strains, while grown in ISP2 liquid media. The growth curve was built in liquid ISP2 culture based on three independently performed cultivations, each of which consisted of three cultivation flasks and each data point was measured three times. Please, review this table in connection to Fig. S3. The μ_{max} for the wild type was calculated to be 0.43 ± 0.08 h^{-1} on average for three cultivations with td of 1.68 ± 0.29 h. Sporulation of the DEL1 and DEL2 strains was estimated for their growth on MS media, where the wild type sporulation was accounted for at $3{,}43 \pm 3{,}05 \times 10^8$ CFU/mL. Wild type sporulation was observed only on MS and ISP4 media, but not ISP2, with an average amount of spores on ISP4 - $2{,}60 \pm 4{,}58 \times 10^8$ CFU/mL, therefore MS media was chosen as the most suitable for growth experiments.

Graph	Strain	μ_{max}	minimal doubling time (td), h	Max OD600	Sporulation, CFU/mL
A, D	WT 1	0.41 ± 0.003	1.69 ± 0.013	8.44	-
A	p056	0.34 ± 0.052	2.11 ± 0.307	3.96	-
B, D	WT2	0.52 ± 0.063	1.34 ± 0.166	6.97	-
B	DEL1	0.55 ± 0.023	1.25 ± 0.05	7.0	$6{,}43 \pm 11{,}15 \times 10^7$
B	DEL2	0.57 ± 0.001	1.22 ± 0.002	7.93	$2{,}83 \pm 3{,}21 \times 10^8$
B	E3I2	0.56 ± 0.031	1.25 ± 0.071	7.56	-
C, D	WT3	0.35 ± 0.012	2.0 ± 0.067	7.09	-
C	p054	0.57 ± 0.026	1.23 ± 0.055	6.84	-
C	p057	0.57 ± 0.03	1.22 ± 0.065	6.44	-
C	p059	0.57 ± 0.006	1.21 ± 0.013	6.69	-

Table S4

Table S4. BLAST hits of genes *tapR1/tpgR1* from *S. rochei* 7434AN4 genome to genes in *S. griseofuscus* genome.

**Gene**	**Hit location**	**Hit name**	**E value**	**Pairwise identity, %**	**Bit score**
tapR1	pSGRIFU1	HEP81_07976	0	100	3993
tapR1	pSGRIFU2	HEP81_08069	0	77.8	1763
tapR1	pSGRIFU3	HEP81_08138	0	71.4	827
tpgR1	pSGRIFU1	HEP81_07977	0	99.8	1002
tpgR1	pSGRIFU2	HEP81_08068	3.48 e^{-161}	82.5	558
tpgR1	pSGRIFU3	HEP81_08137	2.03 e^{-75}	72.1	273

Table S5

Table S5. Summary of all strains, spacers and primers, used in this study.

**Strains, used in this study**		
**Strain**	**Description**	**Source**
E. coli ET12567 pUB307	*dam-13::Tn9 dcm-6 hsdM* Cm^R, pUB8002 helper plasmid	Y. Tong, NBC group, DTU, Denmark
S. griseofuscus pSETGUS	*S. griseofuscus,* carrying pSRTGUS	This work, plasmid pSETGUS received from A. Luzhetskyy, HZI Helmholtz, Germany
S. griseofuscus pKG1139	*S. griseofuscus*, carrying pKG1139	This work, plasmid pKG1139 received from A. Luzhetskyy, HZI Helmholtz, Germany
S. griseofuscus pXJ157-act-int	*S. griseofuscus*, carrying pXJ157-act-int, integrative plasmid that contains *act* gene cluster	This work
S. griseofuscus DEL1 pXJ157-act-int	*S. griseofuscus* DEL1, carrying pXJ157-act-int, integrative plasmid that contains *act* gene cluster	This work
S. griseofuscus DEL2 pXJ157-act-int	*S. griseofuscus* DEL2, carrying pXJ157-act-int, integrative plasmid that contains *act* gene cluster	This work
S. griseofuscus IppsD_1 (p054)	*S. griseofuscus ppsD_1* Trp614Stop Val616Met, base edited BGC 34 strain	(Tong et al; 2020) and this work
S. griseofuscus ItycC_2 (p056)	*S. griseofuscus tycC_2* Ala2643Val Arg2644Stop, base edited BGC 31 strain	(Tong et al; 2020) and this work

S. griseofuscus IHEP81_06602 (p057)	*S. griseofuscus HEP81_06602* Trp221Stop, base edited BGC 30 strain	(Tong et al; 2020) and this work
S. griseofuscus IspkC (p059)	*S. griseofuscus* spkC Trp31Stop, base edited BGC 4 strain	(Tong et al; 2020) and this work
S. griseofuscus E3I2	*S. griseofuscus tycC_7* Trp1566Stop, *spkC* Gly30Ser Trp31 Stop, *ppsE_5* Ala195Val Ser197Phe, multiplexed base edited BGC 1, 4, and 6 strain	This work
S. griseofuscus DEL1	*S. griseofuscus* strain, with pSGRIFU1 and 2 cured	This work
S. griseofuscus DEL2	DEL1 derivative, contains deletion of complete BGC 33, *HEP81_07526–[HEP81_07563]*	This work
**Spacer sequences, used in this study**		
**Name of the spacer**	**Target**	**Sequence, 5′ to 3′**
Sg-ori-del	Replication region of the pSLA2-L like plasmid	CGCTAGCCTCTGATCGC AAA
Sg-BGC33-del	BGC33, *S. griseofuscus*	TACGGCCGGATATCTGT ATT
CBE-Sg-BGC34	BGC34, *S. griseofuscus*	CGCACCGTCCAACCCAG CAG
CBE-Sg-BGC32	BGC32, *S. griseofuscus*	CTCGAGCCACGGGAAC GTAT
CBE-Sg-BGC31	BGC31, *S. griseofuscus*	ACCGCCCGAATAGTCCT TGA
CBE-Sg-BGC30	BGC30, *S. griseofuscus*	GCGATCCAGTCCTCGGT CTT
CBE-Sg-BGC6	BGC6, *S. griseofuscus*	TGCCAGGAGCTTTCCAG GCG

CBE-Sg-BGC4	BGC4, *S. griseofuscus*	TTCCAGCCGTGTTCCAT CGC
CBE-Sg-BGC2	BGC2, *S. griseofuscus*	CCGGTCCACACGTGACC GTT
CBE-Sg-BGC1	BGC1, *S. griseofuscus*	GCCGCGCAGTCCCTGTT GTC
**Primers**		
**Primer name**	**Sequence, 5′ to 3′**	**Purpose**
CW34_rightflank2-rp3-fwd	ggcggcctcggtgagcggggagttcg accgccgaggtccggatct	Right flank for deletion of the entire BGC 33
CW27_cl3-reptemp1-rev	tcagagacagttcatacggtcgcatga actccccgctcaccgagg	Right flank for deletion of the entire BGC 33
CW35_leftflank-rp3-rev	cccacagatccggacctcggcggtcg aactccccgctcaccgagg	Left flank for deletion of the entire BGC 33
CW26_cl3-reptemp1-fwd	ggatctcgtcgaaggcactagaaggg tcgacccagccgtcgttct	Left flank for deletion of cluster 33
CW62_Sg-ori-del-lf-fwd	tcgtcgaaggcactagaaggcaatgg agtccacctcgatg	Left flank for deletion of *S. griseofuscus* pSGRIFU1 plasmid origin
CW63_Sg-ori-del-lf-rev	cgggtgtcgcggacgtgaag	Left flank for deletion of *S. griseofuscus* plasmid pSGRIFU1 origin
CW64_Sg-ori-del-rf-fwd	cttcacgtccgcgacacccggacagc aggggcttcttcac	Right flank for deletion of *S. griseofuscus* pSGRIFU1 plasmid origin
CW65_Sg-ori-del-rf-rev	ggtcgatccccgcatatagggagttca ggcagatcgcgac	Right flank for deletion of *S. griseofuscus* pSGRIFU1 plasmid origin
CW138_sgRNA-CBE-cl2	cggttggtaggatcgacggccgcacc gtccaacccagcaggttttagagctag aaatagc	Primer for ssDNA oligo bridging to knockout BGC 34.
CW139_sgRNA-CBE-cl4	cggttggtaggatcgacggcctcgag ccacgggaacgtatgttttagagctag aaatagc	Primer for ssDNA oligo bridging to knockout BGC 32.
CW140_sgRNA-CBE-cl5	cggttggtaggatcgacggcaccgcc cgaatagtccttgagttttagagctaga aatagc	Primer for ssDNA oligo bridging to knockout BGC 31.

CW141_sgRNA-CBE-cl6	cggttggtaggatcgacggcgcgatccagtcctcggtcttgttttagagctagaaatagc	Primer for ssDNA oligo bridging to knockout BGC 30.
CW142_sgRNA-CBE-cl30	cggttggtaggatcgacggctgccaggagctttccaggcggttttagagctagaaatagc	Primer for ssDNA oligo bridging to knockout BGC 6.
CW143_sgRNA-CBE-cl31	cggttggtaggatcgacggcttccagccgtgttccatcgcgttttagagctagaaatagc	Primer for ssDNA oligo bridging to knockout BGC 4.
CW144_sgRNA-CBE-cl33	cggttggtaggatcgacggcccggtccacacgtgaccgttgttttagagctagaaatagc	Primer for ssDNA oligo bridging to knockout BGC 2.
CW145_sgRNA-CBE-cl34	cggttggtaggatcgacggcgccgcgcagtccctgttgtcgttttagagctagaaatagc	Primer for ssDNA oligo bridging to knockout BGC 1.
CW151_CBE-cl2-check_fwd	gacgctgtgtcccaggacg	Primers to check mutations in BGC 34
CW152_CBE-cl2-check_rev	cgaactcctcggcagggac	Primers to check mutations in BGC 34
CW153_CBE-cl4-check_fwd	cgacgttctcgaccgagtac	Primers to check mutations in BGC 32
CW154_CBE-cl4-check_rev	ctggaaacgccgttctcgc	Primers to check mutations in BGC 32
CW155_CBE-cl5-check_fwd	gtagatcacataggcaggcg	Primers to check mutations in BGC 31
CW156_CBE-cl5-check_rev	tgccgatcgaccctgactac	Primers to check mutations in BGC 31
CW157_CBE-cl6-check_fwd	gttctgctcgctgtgctacc	Primers to check mutations in BGC 30
CW158_CBE-cl6-check_rev	cgagtggctccatggtggtc	Primers to check mutations in BGC 30
CW159_CBE-cl30-check_fwd	acctgtacgtcgaactcgac	Primers to check mutations in BGC 6
CW160_CBE-cl30-check_rev	gagtcgctgctcccgttc	Primers to check mutations in BGC 6
CW161_CBE-cl31-check_fwd	gtgacctgaactcgtagcgg	Primers to check mutations in BGC 4

CW162_CBE-cl31-check_rev	cgtagacggtgatggccttg	Primers to check mutations in BGC 4
CW163_CBE-cl33-check_fwd	cagcggcagtacggtgag	Primers to check mutations in BGC 2
CW164_CBE-cl33-check_rev	ctgcacatcggccggatctc	Primers to check mutations in BGC 2
CW165_CBE-cl34-check_fwd	gcccgacttggagaggatcg	Primers to check mutations in BGC 1
CW166_CBE-cl34-check_rev	ggcaactacgccgacttcac	Primers to check mutations in BGC 1
pXJ-act-t-f	agcaacgatggctgaagtga	To verify the presence of actinorhodin BGC, fwd primer
pXJ-act-t-r	gagcaggtccatgatctcgg	To verify the presence of actinorhodin BGC, rev primer
CW175-plasmid-check_fwd	ccgatgtgaccgtacggatg	To verify the presence of pSGRIFU1
CW176-plasmid-check_rev	ctctcctcatgtactccacgtc	To verify the presence of pSGRIFU1
